# Enhancing professional development in digital STEM education: cross-disciplinary success factors and barriers

**DOI:** 10.3389/fpsyg.2025.1653606

**Published:** 2025-10-08

**Authors:** Mientje Lüsse, Ronja Sowinski, Larissa-Marie Stamer, Simone Abels, Maja Brückmann

**Affiliations:** ^1^Primary Science Education and Social Studies, School I, Department of Educational Sciences, University of Oldenburg, Oldenburg, Germany; ^2^Natural Science Education, School of Sustainability, Institute of Sustainable Chemistry (INSC), Leuphana University Lueneburg, Lueneburg, Germany

**Keywords:** digitalization, science education, ICT skills, out-of-school student labs, digital media education, in-service teacher education

## Abstract

The increasing integration of digital tools into teaching presents opportunities to enhance interactivity, flexibility, and student-centeredness for science education. However, for these opportunities to be fully realized, teachers need to develop the necessary competencies and positive beliefs to effectively incorporate digital media into their pedagogical practices. Therefore, offering high-quality professional development (PD) is essential. Such programs provide teachers with hands-on training, strengthen their self-efficacy, and support student-centered teaching strategies, including the reflective use of digital media. Our ministry-funded project *LFB-Labs-digital* has developed empirically based PD programs in student labs across different subjects. For this context, a design-based research (DBR) approach was conducted within an interdisciplinary quality management (QM) to aim at analyzing specific success factors and barriers of these PD programs. Hereby, we collect data from regular web-based discussions and short follow-up interviews with PD facilitators about their PD programs as well as incorporated observations. The evaluation framework was aligned with established criteria for effective PD, allowing for a systematic analysis of key success factors and necessary modifications. Our findings highlight several key factors for the success of PD programs in student labs. Identified success factors including technical support, curricular alignment, flexible formats, hands-on orientation, peer support and structured reflection opportunities that help teachers critically evaluate and adapt digital strategies to their teaching practice. These factors must be balanced against persistent barriers such as technical and organizational barriers as well as teachers’ heterogeneous digital competencies. Facilitators emphasize the need for PD programs that address diverse teacher needs while maintaining coherence in content delivery. By integrating multiple perspectives—facilitators, and systematic observations—this study contributes to a deeper understanding of how student labs can function as effective PD environments and provides concrete insights for scaling up and optimizing digital competency acquisition across subjects.

## 1 Introduction

The global challenges outlined in the United Nations’ Sustainable Development Goals (SDGs) emphasize the urgent need for high-quality education that enables learners to actively shape a sustainable and digitalized future (ITU, UNDP, 2023). In line with this, the *Digital Education Action Plan 2021–2027* of the European Union highlights the need for education and training systems that effectively foster the development of information and communication technology (ICT) competencies (European Commission, 2023). Within the school context, the integration of digital media and the promotion of related competencies among both students and teachers play a central role in preparing young people for active and reflected participation in digital transformation processes (Koehler et al., 2014).

Digitalization-related competencies include a combination of technical proficiency, pedagogical knowledge, and reflective judgment that enables teachers to purposefully integrate digital tools into their teaching (Koehler et al., 2014; Redecker, 2017). Beyond basic ICT use these competencies encompass skills such as selecting and adapting digital resources to curricular goals, fostering students’ critical engagement with digital media, and creating inclusive learning opportunities (Redecker, 2017). Research has shown that such competencies are not only decisive for teachers’ own classroom practice but also for equipping students with the competencies required for active participation in a digital society (Instefjord and Munthe, 2017; Petko, 2012). Digitalization is particularly relevant in science education, as STEM subjects (Science, Technology, Engineering, Mathematics) increasingly use digital simulations, data collection, and computational tools to model scientific phenomena and support inquiry-based learning (Bewersdorff and Weiler, 2022; Schwedler and Kaldewey, 2020). In this context, teachers need to develop competencies to integrate these tools meaningfully into their lessons, enabling students to engage with authentic scientific practices (Redecker, 2017).

Teachers’ self-efficacy in using ICT has been shown to positively impact their actual use of digital tools in the classroom (Petko, 2012). Consequently, the availability of high-quality professional development (PD) programs that foster self-efficacy and build ICT competencies is of crucial importance (Schulze-Vorberg et al., 2021). Although the demand for digitalization-related PD has increased significantly in recent years, the question remains as to how such programs can be designed to be both effective and sustainable (Bonnes et al., 2022; Schulze-Vorberg et al., 2021). Empirical research has demonstrated that the effectiveness of teacher PD depends not only on its content but also on specific structural and educational design features such as fostering collegial cooperation (Lipowsky and Rzejak, 2015; Schulze-Vorberg et al., 2021). A framework in this regard is offered by Lipowsky and Rzejak (2021), who identified 10 empirically grounded key features of effective PD, ranging from content focus and meaningful activities to providing feedback and an appropriate PD duration. However, little is known about how these features are addressed within PD programs that focus specifically on digitalization and take place in student labs. Student labs, also known as outreach (science) labs (e.g., Kirchhoff et al., 2024a), are usually designed as extracurricular, voluntary, and flexible STEM learning environments where school students engage in inquiry-based activities in authentic laboratory settings (Affeldt et al., 2015; Euler et al., 2015). Digitalization is particularly relevant in student labs, where digital tools such as data collection systems, simulations, and virtual experiments are increasingly used, providing teachers with hands-on opportunities to explore and apply these technologies and supporting the transfer of skills into classroom practice (Lahme et al., 2023). Due to their practical orientation and flexibility, student labs are playing an increasingly important role in pre-service teacher education and have shown promising results in that area (Haatainen et al., 2024; Krofta et al., 2012). However, their potential as settings for in-service teacher PD, particularly in the context of digitalization, has not yet been systematically investigated.

In particular, it remains unclear to what extent PD programs conducted in student labs reflect the established key features of effective PD, and whether there are additional, context-specific factors that influence their design and implementation. Furthermore, even though evidence about the need of digitalization-specific competencies [e.g., Digital Competence Framework for Educators, DigCompEdu by Redecker (2017); and TPACK framework by Mishra and Koehler (2006)] of teachers is known, little empirical evidence is available on the specific barriers that occur when offering digital content and methods in these settings. The present study addresses this research gap by examining digitalization-related teacher PD programs offered in eight German STEM student labs, seven of which are university-based and one is hosted by a museum. These programs were developed and implemented within the research and development project *LFB-Labs-digital* (Kirchhoff et al., 2024b). Based on qualitative data from facilitators and observations, the study investigates how the PD programs address established key features of effective PD, which success factors supported successful implementation, and what implementation barriers arose during the programs.

## 2 Theory

### 2.1 Relevance of digitalization for science education

The ongoing digitalization of education has far-reaching consequences for science teaching and has fundamentally reshaped how knowledge is accessed, constructed, and applied. Digital tools such as simulations, data analysis software, virtual laboratories, and collaborative platforms offer science educators powerful means to support inquiry-based learning, visualize abstract concepts, and connect scientific content with real-world phenomena (e.g., Bewersdorff and Weiler, 2022; Schwedler and Kaldewey, 2020). However, the successful implementation of such technologies does not occur automatically; it requires deliberate pedagogical action. In this context, teachers play a crucial role as mediators of digital transformation in education: They are not only responsible for integrating digital tools into their instruction, but also for fostering their students’ competencies in navigating, critically evaluating, and utilizing digital resources in scientific contexts (Ertmer et al., 2012). As such, teachers are both enablers and gatekeepers of digital learning processes, particularly in disciplines like science where technological tools can significantly enhance conceptual understanding and engagement (Trigo et al., 2023).

Therefore, like their students, teachers need both competencies and positive beliefs regarding the use of digital tools for (content) learning and teaching (Instefjord and Munthe, 2017; Kopcha, 2012; Vogelsang et al., 2019). Regarding the beliefs, however, it is important, that the teachers are able to reflect their own beliefs as well as to make reflected decisions regarding the integration of digital media in class. Such competencies are described within frameworks such as the Technological Pedagogical Content Knowledge (TPACK) model (Koehler et al., 2014; Mishra and Koehler, 2006) and DigCompEdu (Redecker, 2017). Focussing on the three perspectives on teachers’ competencies on content, pedagogical, and technological knowledge, the TPACK model gives an overview about the interplay of the competencies needed. Additionally, the DigComEdu as European framework gives specific examples for teachers on how to develop specific digitally-based competencies. Therefore, these frameworks offer valuable guidance in defining the complex interplay between technological knowledge, subject-specific content, and pedagogical practice, and therefore, describing specifically a digital pedagogical competence. These frameworks highlight the need for an integrated understanding of how digital tools can be meaningfully aligned with instructional goals and scientific epistemologies. For instance, using a simulation to teach chemical reactions is only effective if the teacher can pedagogically scaffold the experience and connect it to underlying scientific principles (Schwedler and Kaldewey, 2020).

In this context, university studies of pre-service teachers are often seen as the place where such competencies are acquired. Teachers’ knowledge of digital media use may be limited or outdated, as their initial university training often took place several years ago and the number of technical innovations increased during the past years (Gudmundsdottir et al., 2014). Thus, the PD (of science teachers) as part of lifelong learning must place a strong emphasis on the cultivation of digital pedagogical competence. Only then it is possible for educators to create learning environments that not only reflect the digital realities of contemporary science, but also prepare students to participate competently and responsibly in a digitally mediated world.

### 2.2 Key features of effective professional development

Against the backdrop of increasing demands on the education system, the PD of teachers has become a central focus of educational policy and academic discourse (Priebe et al., 2019). Teacher PD serves not only the purpose of individual professionalization but also constitutes a key element in school and instructional development. Its relevance spans the entire educational sector: it influences the quality of teaching, the capacity of schools to innovate, and ultimately the educational opportunities of students (Kultusministerkonferenz, 2020). Many countries, including Germany, face structural deficits in PD systems (e.g., Altrichter et al., 2020; Gudmundsdottir et al., 2014; OECD, 2025), which contribute to insufficient ICT competencies among teachers (Fernández-Batanero et al., 2022). Consequently, the need for conducting effective PD programs and analyses of ways to enhance teacher PD has increased in the past years.

**Figure 1:**
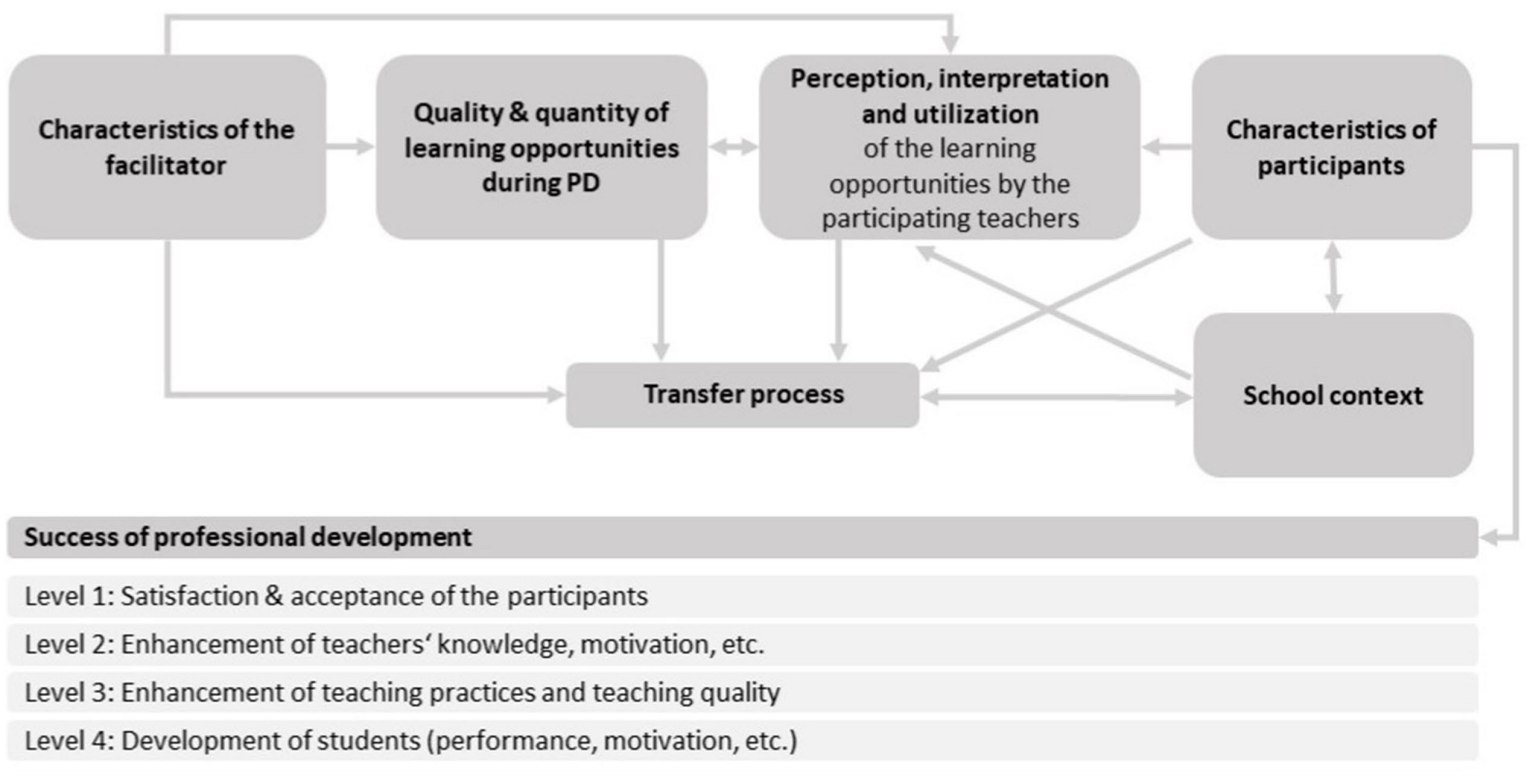
Offer-and-use model for research on teacher PD [adapted from Lipowsky and Rzejak, 2015, p. 30].

Hereby, it is important to get an overview of key features of efficient teacher PD, which was done by different researchers. One model to describe these features is the adapted offer-and-use model by Lipowsky and Rzejak (2015), which builds on earlier work by Lipowsky (2014) and is based on frameworks from instructional research (Figure 1). The central aim of this model is to capture both the provision of PD (“offer”) and the ways teachers engage with and apply the offered content in their school context (“use”). By emphasizing the interaction between these two dimensions, the model underlines that the success of teacher PD depends not only on what is offered, but also on how it is used and how offer and use interact.

Within this model, PD programs can be described in terms of their objectives and design, including of structural and content-related features, the organization of learning activities, and the professional expertise of the facilitators. The 10 key features of effective PD were derived from this model (Lipowsky and Rzejak, 2021) and served as guiding principles for the design of the respective projects within our study (Table 1). However, according to von Sobbe et al. (2025), these features should not be seen as sharply distinct categories, but rather as a flexible orientation framework for describing and analyzing effective PD elements. A balanced combination of Insight, Goal, Technique, and Practice (IGTP) is therefore considered decisive. These four dimensions emphasize the combination of content-related input, clear goal-setting, methodological guidance, and opportunities for practical application (Sims et al., 2021; von Sobbe et al., 2025).

**Table 1:** Ten key features of effective teacher PD by Lipowsky and Rzejak (2021).

Key feature of effective PD	Examples by Lipowsky and Rzejak (2021)
1. Taking into account the research on teaching effectiveness	Design PD in collaboration with educational researchers
2. Focusing on students’ domain-specific processes of learning and understanding	Allow teachers to experience self-directed learning processes themselves
3. Focusing on core instructional challenges	Structure PD around core teaching practices (e.g., the practice of explanation)
4. Content focus	Aim for depth rather than breadth in content coverage
5. Allowing teachers to experience the impact of their pedagogical actions	Highlight teacher–student interactions using tools such as video recordings
6. Cooperation within professional learning communities	Use digital collaboration tools and reflect on their pedagogical potential Address the transfer of PD content explicitly, particularly when teachers are expected to disseminate knowledge within their school
7. Combining and relating phases of input, practice, and reflection	Include recurring cycles of experimentation and reflection
8. Providing feedback to teachers	Encourage the use of multiple feedback sources for deeper insight
9. Appropriate PD duration	Provide relevant content in modular formats to support sustained engagement Offer follow-up support beyond the PD session itself
10. Meaningful content and activities	Emphasize the relevance of the PD topic using concrete examples Ensure that content is transferable to diverse classroom settings

In line with this model, other studies have outlined similar features for effective PD, focusing on the design of the PD program itself (e.g., Daly et al., 2009; Darling-Hammond et al., 2017), the role of the PD facilitators (e.g., Tekkumru-Kisa and Stein, 2017), and the participating teachers (e.g., Richter et al., 2021). While the main focus of this manuscript lies on the design of PD programs, the role of facilitators and the perspective of participating teachers are still acknowledged as important aspects that ultimately influence the success of teacher PD through their interplay.

Regarding the design of the PD program, features like content focus, active learning, collaboration, use of models and modeling, coaching and expert support, feedback and reflection, and sustained duration were also mentioned by Daly et al. (2009), Darling-Hammond et al. (2017), Ertmer et al. (2012), Kopcha (2012), Krille (2020), and Philipsen et al. (2019). However, while such features are well established for PD in general, empirical studies focusing specifically on digitalization-related PD in the natural sciences are scarce. A review by Diepolder et al. (2021) found that existing programs for science teachers remain limited in number and often insufficiently aligned with competency frameworks, highlighting the need for further research and development in this area. With respect to digitalization-related aspects in general, Lipowsky and Rzejak (2021) emphasized that online formats represent a meaningful supplement to effective, multi-part face-to-face training. Digital platforms enable regular, individualized coaching by facilitators, and video materials can be shared and analyzed via secure platforms. In addition, the possibility to get feedback or being part in a peer group receiving mentoring opportunities is highlighted as success factor for a digitalization-related PD (Kopcha, 2012).

The ways in which PD offerings are perceived and utilized largely depend on the individual preconditions of participants (Lipowsky and Rzejak, 2021). These in turn affect the transfer of training content into practice, and thereby the success of the PD. However, contextual factors such as systemic conditions, institutional structures, or regulations remain largely unaddressed in the offer-and-use model. Those factors are outlined for example by Geijsel et al. (2009) and Krille (2020). By doing so, context conditions as the school environment (size, cooperation, or school requirements) and factors of the profession itself which influences the (possibility of) participation on a PD (teaching and work load, or potential class cancelation) are highlighted as important as the individual characteristics of the teachers or the PD program.

Studies on teachers’ PD needs and expectations emphasize aspects such as practice-oriented approaches, the provision or co-development of concrete teaching materials or classroom scenarios, the integration of current (subject-specific) educational research, targeted support measures before and after the PD program, and the inclusion of innovative concepts (Ram Pokhrel and Kumar Behera, 2016; Ropohl et al., 2016). Another frequently mentioned factor is the added value of collaborative work, particularly at the interdisciplinary level through cooperation with colleagues from other STEM subjects (Mumcu et al., 2022).

With regard to digital media, Mumcu et al. (2022) highlight that teachers do not seek training on basic ICT knowledge, but rather on innovative approaches to fostering ICT competencies in teaching and learning processes. Furthermore, teachers express a need for greater support in implementing curricula, especially through access to curated online repositories of teaching materials and digital PD offerings (Hörmann et al., 2024). Flexible and location-independent formats are preferred due to time constraints and long travel distances (Hörmann et al., 2024), although short face-to-face formats such as one-day or half-day workshops are most favored (Brommer et al., 2023).

This preference, however, contrasts with empirical findings showing the high effectiveness of multi-part PD programs (Lipowsky and Rzejak, 2021). Nonetheless, there are indications that teachers would be more willing to participate in extended training formats if these were financially compensated or balanced by a reduction in teaching workload (Hörmann et al., 2024).

### 2.3 Student labs in the context of science education and teacher PD

Student labs are out-of-school learning environments that are primarily applied in STEM education (Affeldt et al., 2015; Hofstein and Lunetta, 2004). A growing body of research has examined the impact of student lab visits on students’ cognitive outcomes and affective responses, including interest, motivation, and attitudes (e.g., Kirchhoff et al., 2022; Molz et al., 2022; Thomas, 2012). Studies show that participation in student lab programs can foster student interest in science and contribute to learning and affective outcomes (Molz et al., 2022). However, findings also indicate that the effects are not necessarily stronger than those achieved through well-designed in-class school lab activities (Kirchhoff et al., 2024a; Molz et al., 2022). This does not imply that student labs are redundant. Rather, they offer valuable and complementary learning opportunities beyond the classroom by providing more authentic experiences and deeper insights into scientific work and laboratory processes that are difficult to simulate in school settings (Kirchhoff et al., 2024a). Neher-Asylbekov and Wagner (2023) emphasize that out-of-school learning environments such as student labs hold great potential to foster scientific interest, particularly among students with limited prior knowledge. However, they also highlight the importance of careful pedagogical and logistical preparation to fully realize this potential.

In Germany, student labs play a significant role in the out-of-school educational landscape. They are primarily operated by universities but also by non-university research institutions, environmental education centers, and other organizations (Euler et al., 2015). Key objectives of student labs in the German context include the promotion of interest and motivation for STEM subjects, the provision of insights into scientific careers, and the development of hard and soft skills through team-based and project-oriented work (Euler et al., 2015; Rieger et al., 2023). Authenticity plays a central role, supported by the proximity to real research environments and the high degree of hands-on experience (Euler et al., 2015).

In addition to their student-centered goals, university-based student labs increasingly involve pre-service teachers in the design and delivery of student lab programs. For example, pre-service teachers guide school groups or develop and test their own instructional concepts in practical seminars embedded within the student lab setting (Krofta et al., 2012; Roth and Priemer, 2020; Schulz et al., 2018). These authentic and flexible environments offer a protected space for experimentation and professional growth. Initial research suggests that active involvement in non-formal learning environments can enhance pre-service teachers’ professional learning and identity development (Haatainen et al., 2024). Additionally, such experiences have been shown to improve pre-service teachers’ digital and digital pedagogical knowledge, as well as their self-efficacy in using digital tools (Martens et al., 2022; Martens and Schwarzer, 2023).

Even though there are indications that teachers who visit university-based student labs with their class can also benefit from the exchange with university members (Schulz et al., 2018), there is still a lack of research on the systematic implementation of digitization-related teacher PD in student labs for in-service teachers. To address this gap, our research study aims to analyze how established key features of effective PD are implemented and potentially expanded within these digitalization-related PD programs in student labs, most of which are located at universities. Additionally, we investigate which barriers may hinder the successful implementation of such PD programs. Specifically, the following research questions are:

RQ1: How are established key features of effective PD by Lipowsky and Rzejak (2021) addressed in digitalization-related PD programs conducted in student laboratories?

RQ2: How do the practice-based success factors identified in digitalization-related PD programs in student laboratories align with, refine, or expand the established criteria for effective PD?

RQ3: What barriers hinder the effective implementation of digitalization-related PD programs?

## 3 Materials and methods

### 3.1 Project structure

This study is embedded in a joint project in Germany entitled “LFB-Labs-digital–Schülerlabore als Ort der Lehrkräftebildung in der digitalen Welt” (LFB-Labs-digital—student labs as a place for teacher training in the digital world), which is part of a federal ministry-funded competence network. Within this project, students’ labs are systematically developed as innovative learning locations for digital teacher PD in the STEM field. These teacher PD focus, for example, on experimental and hypervideos or digital simulations as digital tools, or on examination of the digital habitus of students to further develop model-based learning. It is intended to generate a double impact: on the one hand, qualifying teachers with regard to digitalization-related competencies and, on the other hand, indirectly promoting STEM motivation among students (Kirchhoff et al., 2024b).

The eight PD programs differ in their focused content and digital tools, but all were developed in alignment with key features of effective teacher PD by Lipowsky and Rzejak (2021). This should ensure that the programs systematically incorporate established best practices, while allowing adaptations to fit the specific context. In line with the design-based research approach (Anderson and Shattuck, 2012), the digitization-related PD programs were further developed in iterative cycles of conception, implementation, evaluation, and modification over a period of two and a half years. The aim is to create formats that are both scientifically sound, and practical and implementable.

**Figure 2:**
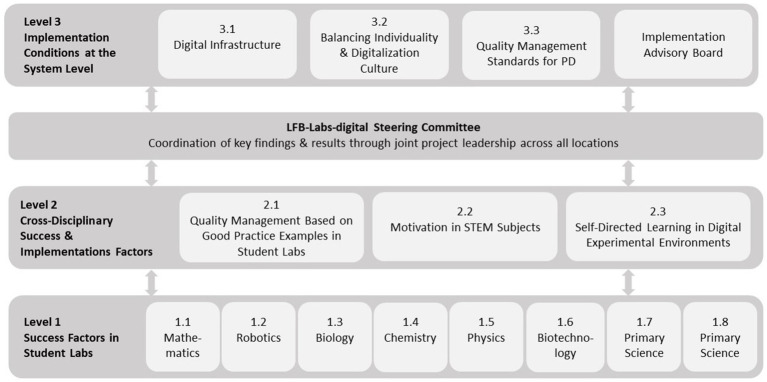
Project structure.

The project structure (Figure 2) shows the subprojects involved and how they are connected within the joint project. Eight STEM student labs are involved in the project, seven of them located in Bielefeld and one in Paderborn, which together form level 1 of the project and offer teacher PD in iterative cycles. The designs of all individual PD programs and the other sub-projects are summarized in Kirchhoff et al. (2024b) and have also been partially presented in individual publications (e.g., Brusdeilins et al., 2024; Kiel and Schwedler, 2023; Lehmenkühler et al., 2024; Ziegler and Stinken-Rösner, 2024).

This article focuses on sub-project 2.1 (Quality Management based on Good Practice Examples in Student Labs). In this sub-project, the offer and use of the PD programs were examined from an interdisciplinary perspective in order to identify success conditions and barriers to implementation. The focus of the presented study is on the offer-side (see offer-and-use-model, Chapter 2.2), i.e., the PD programs provided and the experiences of the PD facilitators.

### 3.2 Methods

To examine the offer-side, a total of eight so-called webtalks as online exchange format were carried out with the PD facilitators within one and a half years. In each webtalk, topics such as transformation, digitalization, good practice, and digital infrastructure were discussed digitally with the PD facilitators of all eight PD programs. The selection of topics was based on the one hand, on the expressed needs and preferences of the PD facilitators. These were regularly collected through short digital surveys before each webtalk, resulted in topics such as teacher recruitment. Using this digital survey, the webtalks were evaluated and refined during the first sessions. Therefore, regarding the design-based research approach, it was possible to elaborate a structure of the webtalks to identify success factors and implementation barriers as good as possible. On the other hand, we aimed to collect data according to our research interests and included topics such as good practice examples and the implementation of key features of effective PD. The sessions initially lasted 1 h but were later extended to one and a half hours in response to the facilitators’ expressed wish for more time to exchange ideas and experiences. In most cases, one or two PD facilitators from each PD program participated the webtalks, so the number of participants was usually between 5 and 10. Moreover, it was possible for other project members (e.g., persons from level 3 subprojects) to take part and share their perspective as well. The researchers of subproject 2.1, who are also among the authors of this study, served as moderators in the webtalks. They prepared evidence-based information on each topic, as well as results from questionnaires within the LFB-Labs project, which were used as interventions during the discussions. For example, insights into teachers’ expectations for the different PD programs were provided based on survey results collected prior to the programs. The facilitators were then asked to reflect on whether these expectations had been addressed in their PD sessions. Finally, results from earlier webtalks concerning the implementation of different aspects in the PD programs were presented to the facilitators, highlighting that most of the mentioned expectations had indeed been addressed. All webtalks were recorded and transcribed, and discussion results were documented via Collaboard or TaskCards by the PD facilitators - both for internal analysis purposes and to ensure transparency for all participants. In addition to the webtalks held online, there was a one-time face-to-face meeting with the PD facilitators. During this meeting, the key contents were documented using TaskCards.

To add the observational data and gain insight into the practical implementation and development processes of the PD programs, short guided interviews were conducted with the PD facilitators across different implementation cycles. The interviews followed a fully structured format and were carried out before and after the first, and second PD run. Hereby, the interview guideline slightly changed between the first interviews before/after the first PD run and in subsequent cycles: For the first interviews three initial questions about the content and structure of their PD programs (“Tell me a bit about what you do in your PD”), the methods they applied (“Which methods do you use in your PD?”), and the degree to which existing lab structures could be utilized or had to be adapted (“Which existing structures could you build on in your lab? What needed to be adapted or transformed?”) were asked. In subsequent cycles, the focus shifted toward reflection on success factors and implementation barriers (“What went particularly well in the last cycle?,” “What challenges did you face during the implementation?”), as well as evaluation of previous goals and planning for the next PD round (“Did you achieve the goals you set?,” “What are your goals for the next round?”). A total of 19 interviews, each lasting between 5:47 and 20:00 min, were conducted with the facilitators of the respective PD programs: four before the first cycle, eight after the first cycle, six after the second cycle, and one after the third cycle.

After four webtalks, and in alignment with the design-based research approach of this study, the webtalk format and topics were reflected within this subproject. In this context, a possible discrepancy between self-reported aspects by the PD facilitators and their actual practical realization was discussed. Some facilitators found it challenging to determine whether they addressed certain of the 10 key features of effective teacher PD. To externally validate the facilitators’ statements of their own PD program, an observation protocol was developed on the basis of the 10 key features of effective teacher PD according to Lipowsky and Rzejak (2021). The protocol was developed on the basis of existing instruments and includes a combination of items: the eight items focused directly on the 10 key features were taken verbatim from the observation tool presented in the IMPRESS project (Rzejak et al., 2023), 11 items were adapted from existing survey instruments on PD quality by the same authors, and eight items were self-developed in reference to concrete suggestions for implementing the key features described by Lipowsky and Rzejak (2021). The protocols included one to four observable items assigned to each key feature. Each item was rated on a three-point Likert scale (1 = low, 2 = medium, 3 = high implementation). In total, six PD sessions were observed, mostly conducted by two researchers collaboratively. The first observed PD session served to pilot the observation protocol. The results (scores) of both researches were compared and differences were discussed (argumentative validation; Creswell, 2007). Based on this process, the observation protocol was revised by adding a section for additional notes. Other revisions were not necessary. Example items of the observation protocol are described in Table 2.

**Table 2:** Example items assigned to the key features of effective teacher PD (Lipowsky and Rzejak, 2021).

Key feature of effective PD (Lipowsky and Rzejak, 2021)	Example items	Source of item
2. Focusing on students’ domain-specific processes of learning and understanding	2.1 The value of digital media for acquiring subject-specific strategies and techniques was highlighted.	Self-developed based on Lipowsky and Rzejak (2021)
	2.2 Self-regulated learning (with digital media) was made tangible for the participating teachers.	
6. Cooperation within professional learning communities	6.1 Opportunities for exchange among participants were provided.	Verbatim from Rzejak et al. (2023)
	6.3 Small teams discussed current questions regarding their own (digital) teaching practices.	Adapted from Rzejak et al. (2023)
10. Meaningful Content and Activities	10.2 Strategies for implementing the (digital) PD content in schools were discussed.	Verbatim from Rzejak et al. (2023)
	10.3 Content related to digital media was developed using case examples.	Adapted from Rzejak et al. (2023)

### 3.3 Data analysis

All qualitative data was analyzed using the method of qualitative content analysis, combining deductive and inductive approaches (Kuckartz, 2018). Webtalks, interviews, and observation protocols were fully transcribed and imported into a single MAXQDA Analytics Pro 2024 project file to enable systematic coding and data management. Once imported, all data sources were treated uniformly in the analysis process.

**Figure 3:**
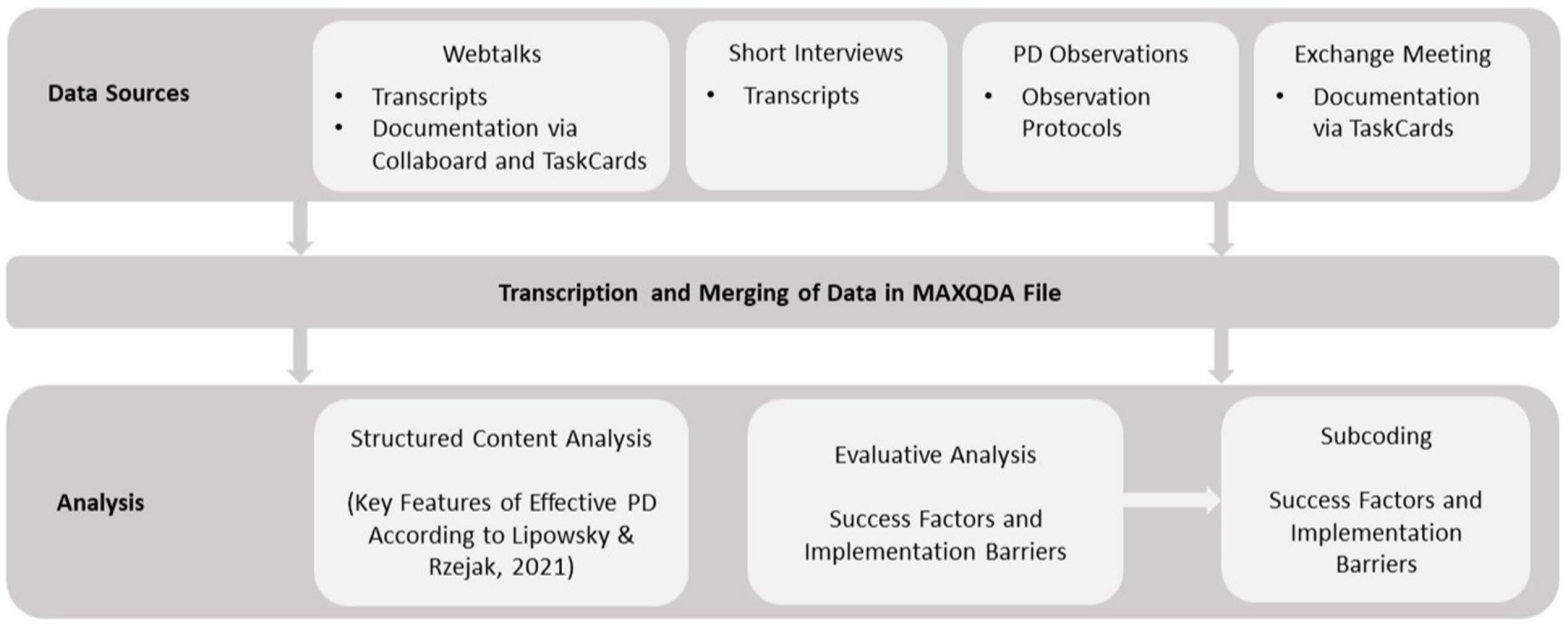
Visualization of the data analysis process.

The analysis followed a dual strategy (see Figure 3). First, a deductive approach was applied to code the transcripts according to the 10 empirically grounded key features of effective teacher PD proposed by Lipowsky and Rzejak (2021). These features served as a theoretical framework for assessing the potential quality and effectiveness of the analyzed PD formats. All statements that could be related to a specific feature were coded, including affirmative, ambivalent, and critical remarks. The deductive coding scheme was documented in a codebook and operationalized using sample quotations prior to the main coding phase.

Second, an inductive approach was used to identify practice-oriented success factors and implementation barriers specific to the use of digital content and formats in student lab-based PD. For this purpose, an additional category system was created to guide the evaluative content analysis. Both the main categories “success factors” and “implementation barriers” were further subcategorized inductively during the coding process. It was agreed that at least one half-sentence would be coded for the analysis, ensuring that each unit is as self-explanatory as possible and represents a coherent thought. For the evaluative analysis, only the statements of the participants in the webtalks were coded and not those of the hosts. To support the development of these subcategories, the AI-assisted coding suggestion tool in MAXQDA Analytics Pro 24.0.0 was used to generate a preliminary set of subcodes. These AI-generated subcodes served as an initial basis for discussion and were subsequently reviewed, revised, and refined by three researchers through communicative validation. All data segments that had initially been coded under the main categories “success factors” and “implementation barriers” were subsequently re-examined and manually re-coded into the refined subcategories, allowing for a more differentiated and thematically coherent analysis.

Observation protocols were analyzed with a focus on open-ended response fields and written comments accompanying individual items. Closed-ended responses (checkboxes) were not included in the qualitative coding process as they primarily served internal quality monitoring purposes.

Transcripts of the interviews and webtalks, as well as the observation protocols, were coded using the same category system to highlight similarities and differences between the facilitators’ perceived perspective and the external perspective on the PD sessions.

To ensure the reliability of the coding procedure, approximately 50% of the dataset was independently coded by two additional trained raters. The resulting intercoder reliability reached *κ* = 0.97 (Kuckartz and Rädiker, 2019). Discrepancies were resolved through additional argumentative validation of the analysis process, ensuring that interpretations were grounded in the data and logically derived from the coding structure (Creswell, 2007).

## 4 Results

In presenting the results, we do not look at each PD program individually, but rather at interdisciplinary findings. Particular emphasis is placed on those results in which aspects of digitalization played a central role or which are relevant when interpreted in the context of digitalization in education.

### 4.1 Implementation of the key features of effective PD in digitalization-related PD programs in student labs

Overall, the key features of effective PD (see chapter 2.2) as outlined by Lipowsky and Rzejak (2021) were addressed in the digitalization-related PD programs conducted in student lab contexts. A comparison between the facilitators’ perspective and the external observations indicates that facilitators tended to report fewer activities related to the 10 key features of effective teacher PD than were actually observed in the PD sessions. For example, only a few facilitators mentioned the importance of a content focus in their PD, whereas such a focus was evident in all observed sessions. In line with RQ1, the analysis examines to what extent and in what form these features appeared across the programs. Concrete examples of how these features were incorporated in the programs are summarized in Table 3. For the key feature appropriate PD duration, the value of 6 (8)/8 in the column “Number of Labs addressing this key feature” reflects that two PD programs were originally designed with a modular structure across several sessions, but were adapted to a one-shot format due to teachers’ time constraints and needs.

**Table 3:** Implementation of the key features of effective PD (Lipowsky and Rzejak, 2021).

Key feature of effective PD (Lipowsky and Rzejak, 2021)	Description	Illustrative quotes	Number of labs addressing this key feature
Taking into account the research on teaching effectiveness	Digital learning presented as a complement to analog learning Theoretical introduction of the topic Early media education as a cross-cutting task in primary schools Emphasis on appropriate selection and targeted use of digital tools	*“[…] we also showed them this model-based investigation, or rather inquiry-based learning, and then there was a lot of, like, that’s way too much preparation and I’d rather just stand at the front of the classroom and hand out worksheets in primary school, and we said, okay, but maybe that’s not (.) the only way to convey knowledge in primary school.”* (Webtalk 02/18/25, Part I, Pos. 62)	7/8
Focusing on students’ domain-specific processes of learning and understanding	Self-directed study by teachers (analogous to students) Use and exploration of the same digital tools as the students	*“This was implemented, for example, by providing teachers with a phase of self-study, during which they also completed the students’ analog tasks, or by using the same digital tools as the students. In doing so, they developed an understanding of the role and significance of learning strategies.”* (Webtalk 02/18/25, Part I, Pos. 8)	8/8
Focusing on core instructional challenges	Observation of students in the student lab; observation and reflection on teacher actions Explicit focus on “explaining” as a core teaching practice	*“They focus on that quite explicitly, for example by addressing explainer videos as a topic before moving on to experimental videos.”* (Webtalk 02/18/25, Part I, Pos. 55)	7/8
Content focus	Concrete topics and themes versus openness and diversity of topics	*“We did not choose an overarching theme for all methods and tried to fit them in, instead, we selected one topic from each area of the curriculum and developed a method for that.”* (Webtalk 02/18/25, Part I, Pos. 42)	8/8
Allowing teachers to experience the impact of their pedagogical actions	Analysis of teacher-student interaction in classroom settings or in the student lab Use of vignettes to analyze student behavior	*“In my professional development program, I actually go to the school with the teachers and conduct a lesson with them. I also observe the teachers. This creates an opportunity to observe and then analyze the interaction between teachers and students.” (Webtalk* 02/14/24, Part I, *Pos. 112).*	5/8
Cooperation within professional learning communities	School-spanning tandems/tridems Peer support Focus on face-to-face formats to foster collegial collaboration Exchange of PD products	*“They work together with us on teaching or testing settings.”* (Webtalk 02/18/25, Part II, Pos. 7)	8/8
Combining and relating phases of input, practice, and reflection	Input phases, in part self-directed Implementation during the PD sessions and in school settings Subsequent reflection	*“Teachers receive both theoretical and practical input, then test out their individual tools in the practice phase, and reflect together in the plenary.”* (Webtalk 02/18/25, Part II, Pos. 50)	8/8
Providing feedback to teachers	Feedback from facilitators and other teachers Support during the phases of input and practice Additional guidance such as click instructions and links to relevant websites	*“We include that in the individual reflection in the interview setting. They reflect, and we also provide feedback and discuss the observations they make or are supposed to make.”* (Webtalk 02/18/25, Part II, Pos. 59)	8/8
Appropriate PD duration	Modular structure of the program Reflection on the time frame and temporal structure	*“And that, I believe, is the reason why teachers told us, ‘I cannot justify being absent for several sessions to my school leadership.’ (.) Even though we actually learned that one-shot trainings are not very effective. But I think that’s where practice and theory clash a bit.”* (Webtalk 02/18/25, Part II, Pos. 84)	6 (8)/8
Meaningful content and activities	Curriculum alignment Concrete connections to teaching practice Use of everyday apps	*“We experience quite clearly that the implementation at the [student] lab, well, I see that as a meaningful activity, the execution of the digital learning unit that they develop is perceived by the teachers very positively and as very enlightening. It seems to be very effective for them to do this, and they are often truly enthusiastic, for example, about the impact it has on student engagement or similar aspects.”* (Webtalk 02/18/25, Part I, Pos. 81)	8/8

**Figure 4:**
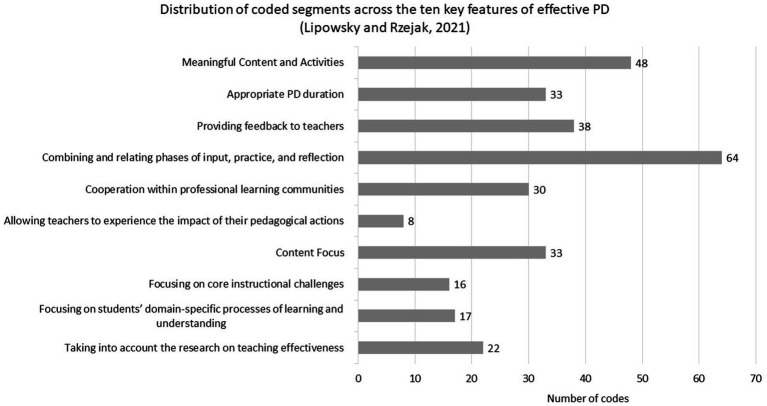
Distribution of coded segments across the 10 key features of effective PD (Lipowsky and Rzejak, 2021) in the data.

Not all eight PD programs addressed all 10 features of effective PD in our data and some PD programs have more data available than others (see Table 3). This variation is partly due to differences in participant engagement during the Webtalks, which resulted in a varying number of statements available for analysis. The distribution of these coded segments is visualized in Figure 4.

Moreover, not all coded segments represent a clear fulfillment of the respective feature, they also include challenges, ambiguities, or indirect references related to the feature. For example, in relation to the first key feature, it was mentioned that the theoretical and scientific background was deliberately kept brief:

“Just from PCK to TPACK and (…) um (…) basically just using that to justify why teachers should engage with digital media. (…) And honestly, the scientific input was very short (…) and you could already tell in the first few minutes that the teachers’ mood started to drop a bit. So I was glad we didn’t dwell on that too much.”(Webtalk 02/18/25, Part I, Pos. 42)

One facilitator emphasized that referring to current research on teaching was especially “*useful for designing the PD itself, meaning for the facilitators*” (Webtalk 02/18/25, TaskCards) and thus tended to be reflected more implicitly in the sessions themselves.

The focus on specific content was implemented quite differently across the PD programs. For example, some programs focused on a particular subject area (e.g., wind energy) and subsequently introduced several digital tools to support the implementation of this topic (Webtalk 02/18/25, Part I, Pos. 48). In contrast, other programs concentrated on one specific digital tool and explored its use across a wide range of topics (Webtalk 09/18/24, Collaboard).

Regarding the promotion of collaboration within professional learning communities, various implementation strategies were evident across the PD programs. Digital tools such as shared documents and collaborative boards were also used to facilitate asynchronous collaboration and the exchange of ideas (Webtalk 06/12/24, Part II, Pos. 28; Webtalk 02/14/24, Collaboard). Multiprofessional team collaboration was particularly encouraged through targeted grouping of teachers from different STEM subjects (Webtalk 02/18/25, Part II, Pos. 18). Face-to-face modules were perceived as especially beneficial to supporting collegial exchange and communication (Webtalk 02/18/25, Part II, Pos. 88).

The PD programs were mostly structured in a modular format, typically consisting of three to four sessions. This number of sessions occasionally led to difficulties regarding teachers’ release from school duties (Webtalk 02/18/25, Part II, Pos. 82) and revealed a tension between theoretical recommendations and practical realities. In contrast, other PD facilitators reported positive feedback indicating that the implementation of a multi-part format combining online and face-to-face sessions was perceived as attractive and “well manageable” (Webtalk 02/18/25, Part II, Pos. 86).

Overall, the duration and scheduling of the PD formats were occasionally adjusted over the program of implementation to flexibly respond to teachers’ needs (Webtalk 02/18/25, Part II, Pos. 8; Interview 01/07/25, Pos. 4). For instance, as mentioned above, two programs originally designed as modular formats across several sessions were adapted to one-shot sessions due to teachers’ time constraints and preferences.

### 4.2 Success factors identified in digitalization-related PD formats in student labs

Based on the qualitative analysis of all data sources, eight categories of success factors were identified that contribute to the effectiveness of digitalization-related PD formats in student laboratories (RQ2). These categories are summarized in Table 4.

**Table 4:** Examples of possible conditions for successful implementation of PD in the context of digitalization-related student labs.

Success factor	Description	Illustrative quotes	Phase
Technical support	Browser-based simulations Availability of school-owned devices Step-by-step guides and quick reference sheets Minimal installation effort required Use of tablets already available in schools	*“Our simulations are all web-based. There’s no need to install anything or push apps to devices.”* (Webtalk 01/28/25, Part I, Pos. 208)	Before, during, after
Targeted teacher recruitment	Direct outreach via mailing lists Personal invitations and network-based recruitment Promotion through free student lab access or material packages Integration of schools with existing digitalization initiatives	*“What worked best, I think, was using our internal mailing list. It includes all the teachers who regularly come to us or have been here before and want to stay informed about our projects. I personally believe this yielded the highest response.”* (Webtalk 05/08/24, Part I, Pos. 16)	Before
Flexible formats	Combination of synchronous and asynchronous elements Mix of online and face-to-face events Optional sessions and individualized participation Integration of students in lab sessions for live observation Digital formats reduce time investment	*“You mentioned release time – we do not need any. The sessions are either asynchronous or scheduled in the late afternoon. Or teachers come to the lab with their own class to experiment – that’s a very attractive setup.”* (Webtalk 05/08/24, Part I, Pos. 16)	During
Curricular alignment	Explicit reference to core curriculum Integration into existing teaching units Use of pre-tested student lab content	*“My impression is that the content becomes meaningful for teachers when they see how it aligns with curricular requirements. They know: ‘Oh, I’ve worked on that before’ or ‘I know where to place this’. That makes it relevant and applicable.”* (Webtalk 02/18/25, Part I, Pos. 84)	Before, during
Low-threshold introduction	No prior knowledge required Gradual and accessible introduction Preliminary familiarization through materials Emphasis on clarity and simplicity	*“I also liked that the first part of the session really made clear: no prior knowledge is assumed, it’s all presented in a very low-threshold way.”* (Webtalk 09/18/24, Part II, Pos. 63)	During
Exchange, feedback and peer support	Regular reflection phases Direct feedback and technical/instructional support Collaborative peer work	*“Teachers work in teams with different subject specializations, so their skills complement each other.”* (Webtalk 02/18/25, Part II, Pos. 18)	During, after
Hands-on orientation	Practical experimentation and tool testing Direct application in the student lab Connection between theory and practice	*“Teachers receive both theoretical and practical input, then test individual tools during the hands-on phase, and reflect on them collectively.”* (Webtalk 02/18/25, Part II, Pos. 50)	During
Applicability	Transfer to classroom settings Ready-to-use teaching materials Support for contextual adaptation	*“And at the same time, we provided them with materials they could use directly in class […] and uploaded them to a shared folder they can access via a link.”* (Webtalk 01/28/25, Part I, Pos. 205)	During, after

The identified success factors proved relevant at different stages of the PD process. *Structural conditions* primarily included the quality of schools’ digital infrastructure, such as the availability of official tablets for teachers (Webtalk 06/12/24, Part I, Pos. 5). From an *organizational* perspective, the flexibility in scheduling the PD sessions was emphasized as crucial, especially when incorporating feedback from teachers regarding their time preferences. For example, adjustments were made by the PD facilitators in course of the PD program such as responding to the request to schedule fixed dates of modular PDs on different weekdays (Webtalk 02/18/25, Part II, Pos. 96).

Among the *content-related factors*, a strong alignment with the curriculum and the practical applicability of the content were highlighted. Particularly beneficial were hands-on elements, such as experimenting in the safe and supportive environment of the student lab (Exchange Meeting 08/22/24, Pos. 17). Opportunities for collaboration among teachers were also mentioned repeatedly, both through joint testing of digital tools (Exchange Meeting 08/22/24, Pos. 28) and through the use of digital platforms like Moodle for communication and exchange (Interview 04/26/24, Pos. 53).

### 4.3 Implementation barriers identified in digitalization-related PD formats in student labs

In addition to the identified success factors, the data sources, particularly the Webtalk transcripts, revealed a number of obstacles that hinder the implementation of PD programs (RQ3). These barriers were systematically coded within the category *Implementation barriers* and are presented in Table 5.

**Table 5:** Implementation barriers in digitalization-related PD programs.

Barrier	Description	Illustrative quotes	Phase
Technical barriers	Internet connectivity issues and missing access to videos/materials Software incompatibility with existing devices (e.g., computer-only, not usable on tablets) Complexity and usability issues with digital tools	*“So we collected the responses [the teachers] just gave, and we heard that the framework conditions are particularly difficult because the equipment is not available.”* (Webtalk 04/10/24, Part II, Pos. 14)	During
Organizational barriers	Difficulties in scheduling and coordination Lack of release time for teachers Difficulties in recruiting sufficient teacher participants	*“So we had the experience that the teachers, um, reported back to us that there were too many dates, three sessions. And, um, that there were also problems with the school because they were not released for more than one session.”* (Webtalk 02/18/25, Part II, Pos. 82)	Before, during
Communication	Limited reach through social media and uncertainty about how to make contact Problems with getting information about the PD to the teachers Hesitation among teachers to raise questions or express problems, especially in online formats	*“There, participants were given the opportunity to receive personal feedback, but that was not really taken up.”* (Webtalk 02/18/25, Part I, Pos. 78)	Before, during, after
Diverse (digital) competencies of the target group	Large differences in teachers’ understanding and handling of digitalization Feelings of insecurity and overload regarding digital elements like simulations or software Heterogeneous learning groups in schools	*“Because I really noticed that […] there is a totally different understanding of digitalization.”* (Webtalk 04/10/24, Part II, Pos. 57)	Before, during
Student-centeredness	Limited involvement of students in the development and testing phase Uncertainty among teachers regarding classroom implementation Need for support during actual implementation	*“So, if I understood this correctly – please correct me if I’m wrong – it’s also about the fact that we do a lot with the teachers in most labs, but very little with the students.”* (Webtalk 02/14/24, Part I, Pos. 112)	During

The barriers identified above occur at different stages of the PD programs. Among the most frequently mentioned are *structural and technical barriers*, such as insufficient digital infrastructure in schools, as well as software and hardware difficulties, e.g., browser tabs freezing or data being lost during browser-based work (Exchange Meeting 08/22/24, Pos. 90–106). *Organizational barriers* were primarily structural and often due to the lack of release time for teachers, which in turn made recruitment and participation difficult. Communication-related issues such as uncertainty over whether schools pass on information to their teachers further complicated participant acquisition (Webtalk 04/10/24, Part I, Pos. 7). In addition, not all teachers were equally comfortable with digital communication formats, which may have negatively impacted interaction during the sessions (Webtalk 02/18/25, Part II, Pos. 91).

*Content-related barriers* were particularly linked to the heterogeneity of teachers’ prior knowledge, especially concerning digital competence. Not only differs the teachers’ prior knowledge of digital media, but the students’ different prior knowledge must also be taken into account, which makes selecting appropriate digital tools complex (Webtalk 06/12/24, Part I, Pos. 5). Although the involvement of students in the PD programs took place through student lab visits, there could have been even more focus in some PD programs in order to analyze the students’ perspectives and the teacher-student interaction even more. This also relates to the observation that teachers often apply the training content independently in their classrooms, making it difficult to evaluate implementation when facilitators are not present (Webtalk 02/14/24, Part I, Pos. 112).

## 5 Discussion

### 5.1 Implementation of the key features of effective PD in digitalization-related PD programs in student labs

The 10 key features of effective PD as outlined by Lipowsky and Rzejak (2021), offer a valuable framework for designing and analyzing PD programs and align in many aspects with other studies on effective PD (e.g., Darling-Hammond et al., 2017; Desimone and Garet, 2015; Kopcha, 2012; Sims et al., 2021). In this study, all PD programs were explicitly designed with these 10 key features in mind, ensuring that the structure, content, and implementation strategies reflected the principles outlined by Lipowsky and Rzejak (2021). These features already include many examples of how digital tools can support teaching and learning, such as digital resources for promoting students’ cognitive activation (key feature 1). Similar examples were also observed in the PD programs analyzed in our study. In some cases, our findings even extend the examples given by Lipowsky and Rzejak (2021). For instance, the core teaching practice of explaining was implemented using teacher-produced explainer videos (Observation Protocol 02/05/25, Pos. 103). The integration of input, practice, and reflection (key feature 7) was also addressed in the PD programs, although in highly diverse formats. Some phases (e.g., input) were delivered asynchronously and online, while others (e.g., hands-on activities in the student lab) took place in person. These implementation formats varied not only between different PD programs but sometimes even across different iterations of the same program (Interview 04/26/24, Pos 63). In this context, there is a discrepancy between the flexibility afforded by digital formats and the desire for face-to-face interaction, particularly in fostering collegial collaboration. While digital formats allow for greater scheduling flexibility (Webtalk 06/12/24, Part II, Pos. 14), teachers also emphasized the benefits of face-to-face interaction for meaningful professional exchange and collaboration (Webtalk 02/18/25, Part II, Pos. 86).

A further field of tension emerged with regard to the duration of PD programs. Although multi-part and longer-term PD formats are generally considered more effective (Lipowsky and Rzejak, 2021), PD providers reported that some teachers were only granted leave for one session, making a series of three sessions difficult to implement (Webtalk 02/18/25, Part II, Pos. 82). In contrast, other participants appreciated the longer duration and found mixed formats (e.g., partially online, partially in person) both useful and feasible (Webtalk 02/18/25, Part II, Pos. 86). These findings underscore the importance of tailoring PD duration not only to teachers’ prior knowledge and experience, as Lipowsky and Rzejak (2021) emphasize, but also to their available time and structural conditions (Hörmann et al., 2024).

Overall, it became evident that the 10 key features cannot always be clearly defined in our practice. For example, providing post-training support is assigned to key feature 9 (appropriate PD duration) by Lipowsky and Rzejak (2021), but in our study, this was also coded under key feature 8 (feedback and support). The features should therefore not be viewed as sharply distinct categories, but rather as a flexible orientation framework for describing and analyzing effective PD elements. In line with the considerations of von Sobbe et al. (2025) and Sims et al. (2021), it also shows that it is not the complete fulfillment of all 10 key features in each PD program that is decisive, but rather a balanced combination of Insight, Goal, Technique, and Practice (IGTP). However, it is important to acknowledge that this study does not directly measure the effectiveness of the PD programs. Effectiveness or success may be measured through different outcomes, such as teacher knowledge gain, changes in instructional practices, teacher self-efficacy, or student learning outcomes (e.g., Darling-Hammond et al., 2017; Sims et al., 2025). Therefore, the results primarily describe the extent to which the key features of successful PD (Lipowsky and Rzejak, 2021) have been addressed in the PD programs, but do not allow for direct conclusions about their impact.

### 5.2 Potential success factors and implementation barriers identified in digitalization-related PD programs in student labs

The analysis of digital PD programs in student labs revealed several potential success factors considered essential for effective implementation. While many of the identified success factors and barriers align with general findings from research on effective PD, the student lab setting offers unique affordances for hands-on exploration, authentic experimentation, and direct engagement with digital tools alongside students, which may enhance the practical applicability of PD content.

*Technical support* was described as a prerequisite for success in all phases of the PD program. It concerns both the structural level (e.g., availability of school-owned devices) and the PD level (e.g., browser-based tools, step-by-step guides). The importance of infrastructural support has been highlighted in earlier studies on digital PD (Daly et al., 2009). Technical support regarding the availability and accessibility of digital tools aligns with DigCompEdu’s emphasis on selecting, creating, and managing digital resources effectively (Redecker, 2017).

The structural level also has a major influence on *teacher recruitment* as teachers usually need time off from school leaders to take part in PD programs. At the PD level, teacher recruitment can be facilitated for example through direct outreach and network-based communication. This factor plays a crucial role prior to the PD implementation and was considered essential for reaching interested and engaged participants. *Flexible formats*, such as a mix of synchronous and asynchronous elements allowed for individualized engagement and reduced time constraints. The scope and timing of the PD programs were adjusted in some cases in order to meet the needs of the teachers. This aspect essentially corresponds to the design feature *appropriate PD duration* described by Lipowsky and Rzejak (2021) and is in line with the discrepancy between empirical findings and teachers’ needs described in the state of research. Similar barriers have also been reported in other projects offering teacher PD in Germany (Sowinski et al., 2025). Other studies also emphasize the need for flexible offers in terms of space and time (Bonnes et al., 2022; Hörmann et al., 2024). *Curricular alignment* was another frequently mentioned factor. This category refers to PD content that aligns closely with the school curriculum, making it easier for teachers to identify where and how to implement new ideas in their own practice. Such alignment has been described as an important feature of effective PD in previous research (e.g., Lipowsky and Rzejak, 2021).

On a content-related level, given the varying levels of prior knowledge regarding digital technologies among teachers, a *low-threshold introduction* emerged as a key feature. This can be related to TPACK’s emphasis on integrating technological knowledge with pedagogical and content knowledge (Mishra and Koehler, 2006), addressing different prior knowledge levels of teachers. Such an introduction included clear communication that no prior expertise was required, and the use of easily accessible materials to support familiarization. Another frequently emphasized success factor was structured *exchange, feedback and peer support*, which aligns with the criterion of providing feedback to teachers (Lipowsky and Rzejak, 2021). Collaborative phases and feedback were integrated into the PD process, and in some cases, digital platforms (e.g., Moodle) were used to extend this exchange beyond the formal PD sessions. These digital platforms for structured peer exchange and feedback reflects the dimension of professional engagement and collaboration in DigCompEdu (Redecker, 2017), highlighting the importance of teachers’ active participation in professional learning communities.

The use of the student laboratory as an experimental learning environment could foster a strong *hands-on orientation* as teachers were able to explore digital tools in a practical setting, sometimes alongside students. Such active learning formats are widely considered a key element of high-quality PD (e.g., Darling-Hammond et al., 2017; Fernández-Batanero et al., 2022). Immediate *applicability* was supported by the provision of ready-to-use teaching materials and opportunities for direct transfer into classroom settings. While not addressed as a standalone feature in Lipowsky and Rzejak (2021), this aspect was mentioned across multiple cases in this study. In addition to the potential success factors, several implementation barriers were identified in our digitalization-related PD programs that can be directly related to the success conditions discussed above. *Technical barriers*, such as insufficient digital infrastructure in German schools, have also been critically highlighted by Lorenz et al. (2022), as they can hinder the integration of digital tools into teaching. The success factor *Technical Support* directly corresponds to this barrier and illustrates possible approaches to overcoming it, such as the use of “browser-based versions as a practical alternative to apps” (Exchange Meeting 08/22/24, Pos. 13–14).

*Organizational barriers*, such as the difficulty of attending PD programs due to the lack of official release from teaching duties, are also reported in previous studies (Hörmann et al., 2024; Krille, 2020). General time constraints are frequently cited as a key obstacle to participation there. In this context, Krille (2020) further emphasizes that insufficient or poorly disseminated information about available PD opportunities constitutes an additional barrier. It therefore warrants further investigation how teachers access information about PD opportunities, how they process it, and what kind of information they actually require. In our study, the provision of transparent and targeted information as part of a teacher-specific recruitment strategy emerged as a promising success condition.

A core *content-related barrier* in designing digitalization-related PD is the heterogeneity of teachers’ digital competencies. In the primary education sector in particular, mathematics teachers in Germany participate in ICT-related training significantly less frequently than the international average (24.0% versus 41.7% within the 2 years prior to TIMSS 2023 survey; Schwippert et al., 2024), although participation rates have increased considerably in recent years (8.4% in TIMSS 2019 survey; Schwippert et al., 2020). At the same time, the German teachers surveyed felt less competent with regard to the use of digital media than the teachers surveyed in Austria and Switzerland (Huber et al., 2020). In response to this, our findings highlight the importance of a *low-threshold, target group-specific introduction* within the PD program. In LFB-Labs-digital, the recruitment material for teachers explicitly emphasized that no prior digital knowledge was required (Webtalk 09/18/24, Part II, Pos. 63). Beyond knowledge acquisition in digital media, the pedagogical dimension must not be neglected (Fernández-Batanero et al., 2022). This is also reflected in our findings, as insufficient integration of student perspectives into the design of the PD was identified as a further implementation barrier. Offering all participating teachers a (free) visit to the student lab with their own school class may help to bridge this gap and represents a further potential success factor.

An overview of the identified implementation barriers, corresponding success conditions, illustrative examples, and resulting implications is provided in Table 6.

**Table 6:** Overview of implementation barriers, corresponding success factors, and implications for digitalization-related PD in student labs.

Barrier	Example	Corresponding success factors	Example	Implication
Technical barriers	Software incompatibility with existing devices	Technical Support	Browser-based simulations	Technical feasibility should be supported through anticipatory planning and the provision of accessible devices.
Organizational barriers	Difficulties in recruiting participants	Targeted teacher recruitment Flexible formats Curricular Relevance	Use of existing school networks Flexible scheduling	Recruitment and scheduling strategies should consider teachers’ time constraints and curricular needs.
Communication	Reluctance to express problems, especially in online formats	Exchange, Feedback and Peer Support	Collaborative peer work	Safe spaces for open exchange should be created to foster engagement and support the implementation process.
Diverse (digital) competencies of teachers	Differences in teachers’ understanding of digital tools	Low-Threshold Introduction Exchange, Feedback and Peer Support Technical Support	Step-by-step guides	Differentiated and supportive formats should be designed to enable meaningful participation for teachers with varying levels of digital competence.
Student-centeredness	Limited involvement of students	Hands-on Orientation Applicability	Transfer to classroom settings	Authentic classroom connections and the inclusive involvement of all students in the student lab setting could enhance the relevance and sustainability of the PD.

### 5.3 Limitations and future research

This study is mainly based on qualitative data from webtalks with PD facilitators, supplemented by observation protocols. Several limitations arise that should be considered when interpreting the findings. A central limitation is that only statements explicitly made during the webtalks could be considered and coded. As a result, the data is limited to what was verbally expressed and implicit or unspoken aspects could not be captured. Additionally, there is a potential bias due to the thematically pre-structured nature of the webtalks: participants were specifically asked about the 10 key features of effective PD (Lipowsky and Rzejak, 2021), for example through guiding questions such as “How do you implement these features in your PD?” or “What are the challenges in implementation?.” This could have steered responses in a particular direction and led to selective focus of the discussion.

Another limitation lies in the subjectivity of the PD facilitators’ statements. To address this, we supplemented the facilitator statements with observation protocols and short guided interviews. The observation protocols, for instance, provided additional insights into how selected features of effective PD were realized in practice, while the interviews captured facilitators’ reflections across the implementation cycles. Nevertheless, it must be acknowledged that the assessments of how the key features were implemented are largely based on self-reports and may therefore be subjectively biased. Therefore, the present study mainly reflects the offer-side perspective of PD facilitators. Future research should therefore also include the user perspective, i.e., how participating teachers perceive and make use of the PD programs. Complementing facilitator perspectives with additional data sources, such as teachers’ perceptions, classroom observations, and student learning outcomes, would allow a more comprehensive and less biased assessment of how PD features are actually enacted and received in practice. A corresponding study focusing on teachers’ reception and evaluation of these PD programs is currently in progress.

In the process of developing subcategories, an AI-assisted coding tool was used to generate initial suggestions. While this approach supported efficiency and transparency, it also has inherent limitations, such as the risk of context-insensitive code proposals or algorithmic bias. These limitations were mitigated by involving three researchers in the code review and refinement process.

Our study takes an interdisciplinary approach and considers eight different PD programs across various STEM subjects. The results therefore do not allow conclusions about the specific design of individual PD programs. Future research could systematically examine which features are particularly well or poorly implemented under which conditions in digitalization-related PD in student labs, and how subject-specific differences play a role.

Another aspect that could only be addressed peripherally in this study concerns the sustainability of PD effects. While references were made to supplementary materials, networking opportunities, or follow-up mechanisms after the PD programs, a systematic investigation of long-term effects was not conducted. In this context, the influence of individual characteristics of PD facilitators appears to be a promising research focus, as has already been explored in some initial studies (Lipowsky and Rzejak, 2021). Investigating the role of facilitator characteristics in digitalization-related PD in student labs, especially in light of sustainable implementation of PD content, could therefore play an important role in future research (cf. Stamer, 2025).

## 6 Conclusion

Digitalization-related PD programs in student labs offer promising opportunities to foster digital competencies among teachers through hands-on, practice-oriented learning in authentic settings. Our findings show that the key features of effective PD by Lipowsky and Rzejak (2021) can be implemented in student lab contexts, sometimes with adaptations that reflect the specific affordances and challenges of digital tools and hybrid learning environments. Rather than rigidly applying all 10 key features, our results suggest that effective PD in digitalization contexts depends on a balanced and context-sensitive combination of content input, goal clarity, practical application, and methodological support (cf. IGTP model, Sims et al., 2021; von Sobbe et al., 2025). We note that this finding is based on our analysis of digitalization-related PD programs conducted in student labs, a setting that is still rare, and therefore cannot be generalized to all types of PD programs. Future research should further investigate whether these insights apply in other contexts. Moreover, the results are derived from the facilitator and researcher perspective, and future research should complement this with the user perspective, i.e., participating teachers’ perceptions and classroom outcomes, to comprehensively assess the PD programs’ effectiveness.

Our study identifies a set of overarching success factors, such as flexible formats, technical support, curricular relevance, low-threshold introduction within the PD program, and structured peer exchange that could be crucial for meaningful implementation. These align closely with, and sometimes extend, the established features of effective PD. Importantly, they must be viewed in relation to corresponding implementation barriers, including technical and organizational barriers, and heterogeneous digital competencies among teachers.

Future research should investigate how sustainable learning outcomes can be supported beyond the PD programs. This includes examining the long-term transfer of digital competencies into classroom practice, potential effects on student learning, and the durability of teachers’ instructional changes. Particular attention should be given to the role of facilitators, including their content and pedagogical expertise, their ability to foster reflective dialog, and their responsiveness to participants’ needs (Lipowsky and Rzejak, 2019, 2021). In addition, subsequent studies should consider contextual factors such as school structures, collegial support, and institutional policies that may facilitate or hinder the implementation and transfer of PD content. Longitudinal studies may offer deeper insights into how digital PD programs in student labs can contribute to long-term professional growth and the development of adaptive facilitation practices.

## Data Availability

The original contributions presented in the study are included in the article/supplementary material, further inquiries can be directed to the corresponding author.
